# Genetic Alterations Predict Long-Term Survival in Ductal Adenocarcinoma of the Pancreatic Head

**DOI:** 10.3390/cancers14030850

**Published:** 2022-02-08

**Authors:** Sami-Alexander Safi, Lena Haeberle, Wolfgang Goering, Verena Keitel, Georg Fluegen, Nikolas Stoecklein, Alexander Rehders, Wolfram Trudo Knoefel, Irene Esposito

**Affiliations:** 1 Department of Surgery (A), Medical Faculty, Heinrich-Heine-University, University Hospital Duesseldorf, 40225 Duesseldorf, Germany; sami-alexander.safi@med.uni-duesseldorf.de (S.-A.S.); Georg.Fluegen@med.uni-duesseldorf.de (G.F.); Nikolas.Stoecklein@med.uni-duesseldorf.de (N.S.); Rehders@med.uni-duesseldorf.de (A.R.); Knoefel@hhu.de (W.T.K.); 2 Institute of Pathology, Medical Faculty, Heinrich-Heine-University, University Hospital Duesseldorf, Moorenstr. 5, 40225 Duesseldorf, Germany; LenaJulia.Haeberle@med.uni-duesseldorf.de (L.H.); Wolfgang.Goering@med.uni-duesseldorf.de (W.G.); 3 Department of Gastroenterology, Hepatology and Infectious Diseases, Medical Faculty, Heinrich-Heine-University, University Hospital Duesseldorf, 40225 Duesseldorf, Germany; Verena.Keitel@med.uni-duesseldorf.de

**Keywords:** NGS, *KRAS*, *TP53*, PDAC, pancreatic cancer, mutation

## Abstract

**Simple Summary:**

Pancreatic cancer is notorious for its poor prognosis. However, rare long-term survivors of pancreatic cancer exist. The aim of this study was to characterize the molecular profile of pancreatic cancer long-term survivors, to improve the stratification and management of pancreatic cancer patients in the future. Thirty-nine pancreatic cancer patients including short-term and long-term survivors were evaluated thoroughly. Their molecular profile was analyzed using panel next generation sequencing. As a result, patients with mutations commonly found in pancreatic cancer (*KRAS* G12D mutations and/or *TP53* nonsense and splice site mutations) showed significantly worse survival. In contrast, long-term survivors of pancreatic cancer did not show the above-mentioned mutations but did show rare mutations of *KRAS* (Q61H/D57N). In conclusion, long-term survivors of pancreatic cancer do have a distinct molecular profile. Further studies using larger patient cohorts are warranted to confirm these results and possibly unravel rare potential targets for targeted therapy in pancreatic cancer.

**Abstract:**

Background: Survival of patients with adenocarcinoma of the pancreas (PDAC) is poor and has remained almost unchanged over the past decades. The genomic landscape of PDAC has been characterized in recent years. The aim of this study was to identify a genetic profile as a possible predictor of prolonged survival in order to tailor therapy for PDAC patients. Methods: Panel next generation sequencing (NGS) and immunohistochemistry (IHC) were performed on paraffin-embedded tumor tissues from curatively treated PDAC patients. Tumor slides were re-evaluated with a focus on the histomorphology. Patients were subgrouped according to short and long overall (<4 years/>4 years) and disease-free (<2 years/>2 years) survival. Results: Thirty-nine patients were included in the study. Clinicopathological staging variables as well as the histomorphological subgroups were homogenously distributed between short- and long-term overall and disease-free survivors. In survival analysis, patients with the *KRAS* G12D mutation and patients with *TP53* nonsense and splice-site mutations had a significantly worse overall survival (OS) and disease-free survival (DFS). Patients with long-term OS and DFS showed no *KRAS* G12D, no *TP53* nonsense or splice-site mutations. Rare Q61H/D57N *KRAS* mutations were only found in long-term survivors. The allele frequency rate of *KRAS* and *TP53* mutations in tumor cells was significantly higher in short-term disease-free survivors and overall survivors, respectively. Conclusions: NGS of PDAC revealed significant differences in survival outcome in a patient collective with homogenously distributed clinicopathological variables. Further multi-institutional studies are warranted to identify more long-term survivors to detect genetic differences suitable for targeted therapy.

## 1. Introduction

Ductal adenocarcinoma of the pancreatic head (hPDAC) is characterized by a poor prognosis with a median overall survival of approximately 6 months, and is predicted to become the second leading cause of cancer-related death in the United States and also in Germany by 2030 [1,2]. To date, the only curative therapy remains margin-negative oncological resection with an adjuvant treatment regime starting within 6 weeks after surgery [3,4]. PDAC metastasizes primarily to the peritoneum, liver and lung [5,6]. At diagnosis, 50% of patients already have distant metastases, and a further 30% present with locally advanced disease and are therefore also not eligible for surgery. Thus, only 20% of patients with PDAC receive surgery with curative intent. Taken together, PDAC is regarded as one of the most lethal cancers [7,8].

Although therapeutic regimens have been modified over the past decades, and a detailed pathological evaluation of the resection status has been implemented, survival outcome has not significantly changed over the past years [9,10,11,12]. The yet not entirely understood molecular alterations in hPDAC might be responsible for the lack of effective chemotherapeutic drugs, and thus therapy resistance and tumor recurrence. hPDAC is a highly heterogeneous disease. Histomorphological and molecular subtypes of hPDAC have recently been discovered, and their importance as possible indicators of prognosis and therapy response after adjuvant therapy is being investigated. In 2008, Jones et al. reported the first genome-wide analysis of PDAC [13], whereby the driver function of previously known genes, such as *TP53* and *SMAD4*, was confirmed [14,15,16]. Genetic factors relevant for PDAC prognosis, such as certain mutations or number of mutated driver genes, have been reported in the literature [17]. However, specific factors associated with the long-term survival of patients with hPDAC are still poorly understood.

Previous studies on the survival of patients with surgically resected hPDACs have reported that smaller tumor size, negative lymph nodes and margin clearance are predictors of a more favorable prognosis, but similar features are found in short-term survivors as well [12,18,19,20,21,22]. Thus, additional factors must play a supplemental role in determining long-term survival in patients with hPDAC. For example, an altered tumor microenvironment or enhanced immune response dependent on tumor-produced neoantigens have been shown to play an important role in long-term survival of hPDAC [23]. We therefore aimed to analyze whether any differences exist in the mutational profile of short- and long-term survivors of PDAC.

## 2. Materials and Methods

### 2.1. Patient Selection and Clinicopathological Data

Patients who received surgery for hPDAC between 2003 and 2016 at the University Hospital of Duesseldorf were included. Exclusion criteria were patients with (1) malignancies of the pancreatic head other than PDAC, (2) TNM staging without information of lymphatic, perineural and venous invasion (Lx, Pnx, Vx), (3) resection status evaluation without CRM implementation, (4) insufficient follow-up information, (5) patients who succumbed within 30 days of surgery, and (5) patients who did not receive a complete course of an adjuvant treatment regimen. Clinicopathological data (gender, age at the time of surgery, disease-free survival (DFS) overall survival (OS), and results of follow-up examinations including time of diagnosis of metastases and site of metastases) were retrieved and collected from patients’ medical records. The data were analyzed retrospectively. In order to evaluate disease-free survival (DFS), all lesions highly suspicious of metachronous disease detected on imaging were considered in the survival analysis. Furthermore, relapse-specific survival (RSS), defined as the survival from the date of the relapse diagnosis until death, was calculated.

Data on the TNM categories (size of tumor/involvement of adjacent arteries, lymph node status and status on distant metastasis), along with grading, lymphatic, venous invasion and perineural invasion were retrospectively collected from the original histopathological reports for each patient. The TNM staging system, if applicable, was updated to the 8th Edition of the UICC TNM classification of malignant tumors [24].

Histopathological slides obtained from the tumor center were re-evaluated. Only cases with enough available tumor material were included in the analysis. Furthermore, all tumor slides were re-evaluated for histopathological subtyping according to the newest WHO classification [25].

### 2.2. Histopathological Slide Preparation and DNA Isolation

Five 4-µm slides were cut from one representative paraffin block. One slide was stained with hematoxylin and eosin and used for the final histopathological evaluation. The tumor area was marked and DNA isolation was performed using the Qiagen™ GeneRead DNA FFFPE Kit™ (Qiagen™, Hilden, Germany) according to the manufacturer’s instructions (Supplemental Figure S1).

### 2.3. DNA Sequencing

Extracted DNA was first quantified by Qubit™ dsDNA BR Assay Kit (Thermofisher, Darmstadt, Germany). Subsequently, quantitative PCR (qPCR) was performed employing a custom primer assay (HML-2 for: 5′-AAACGCCAATCCTGAGTGTC-3′; HML-2 rev: 5′-CATAGCTCCTCCGATTCCAT-3′) directed against a subset of long terminal repeats (LTRs) from HML 2 human endogenous retroviruses with Power SYBR™ Green PCR Master Mix on a StepOnePlus™ real-time PCR system (both Thermofisher, Darmstadt, Germany).

### 2.4. Library Preparation and Massive Parallel Sequencing

Library preparation was carried out using Ion AmpliSeq™ Library Kit 2.0 and Ion AmpliSeq™ Cancer Hotspot Panel v2 with 10 ng of amplifiable DNA following manufacturer’s recommendations. Ion Xpress™ barcode adapters kits were utilized for barcoding the libraries. Afterwards, libraries were quantified by qPCR using the Ion Library TaqMan™ quantitation kit on a StepOnePlus™ real-time PCR system and were compiled equimolarly for subsequent sequencing reaction. Massive parallel sequencing was conducted on an Ion S5 system using the Ion 520™ and Ion 530™ Kit-OT2 with an Ion 530™ chip. Primary data analyses were performed by Ion Torrent Suite software. For variant annotation, generated binary alignment map (BAM) files were uploaded to and analyzed by Ion Reporter™ software using recommended analysis parameters for the Ion AmpliSeq™ Cancer Hotspot Panel v2. Detected variants were examined using the Integrative Genomics Viewer (IGV) [26,27]. All reagents and software were from Thermofisher (Darmstadt, Germany).

### 2.5. Immunohistochemistry

Tumor tissue slides were stained for p16, p53 and Smad4 on the Ventana BenchMark ULTRA automated IHC/ISH slide staining system (Roche Diagnostics, Tucson, AZ, USA) according to the protocols established for routine diagnostics at the Institute of Pathology of the Heinrich Heine University and the University Hospital Duesseldorf, Germany (Supplemental Table S1). Staining was analyzed in a blinded fashion for clinicopathological data by two independent investigators (S.A.S. and L.H.). For p16 and Smad4, only complete lack of staining was classified as loss of expression. For p53, either complete loss of expression (no staining) or overexpression (strong expression in >75% of tumor cells) was classified as aberrant expression (Supplemental Figure S2).

### 2.6. Statistical Analysis

The Wilcoxon test was used to analyze the differences in clinicopathological parameters and mutations. The Mann–Whitney U test was used to examine numerical data and to correlate the distribution between clinicopathological variables. For categorical data, the Chi-square test and the Pearson’s test were applied. Overall survival (OS), disease-free survival (DFS) and relapse-specific survival (RSS) were included for outcome measures. OS was determined as the period from the date of surgery until the date of death of any cause or until the date of last follow-up. DFS was defined as the period from the date of surgery until the date of diagnosed metachronous metastases or local recurrence. RSS was the period from the date of recurrence until death or last follow-up. Kaplan–Meier curves were generated and analyzed using the log-rank (Mantel–Cox) test. Hazard ratios (HRs) with 95% confidence intervals (CIs) were estimated. Analyses were performed using SPSS^®^ statistics for Windows (version 26.0; SPSS, Inc., Chicago, IL, USA). *p* < 0.05 was considered to indicate a statistically significant difference.

The analysis was performed in conformity with the Declaration of Helsinki and the rules of good clinical practice. Furthermore, a positive vote of the Institutional Review Board (IRB) of the Ethics Committee, Heinrich Heine University Duesseldorf (IRB-no. 3281 and 5387) was obtained.

## 3. Results

### 3.1. Histopathological and Genetic Analysis

**Figure 1:**
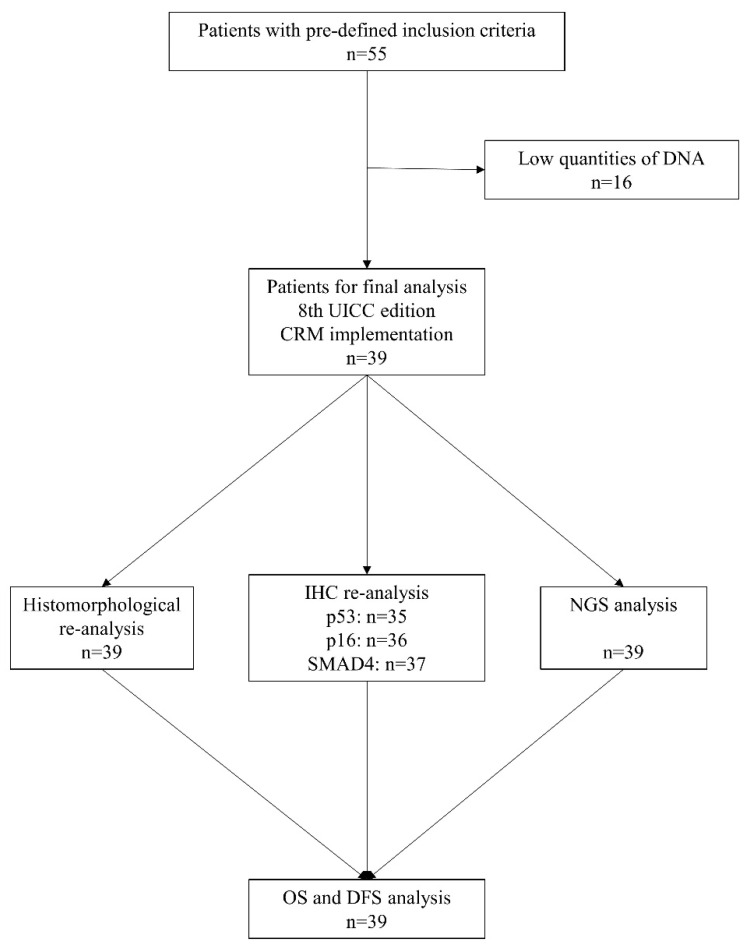
Flow chart of study cohort (CRM: circumferential resection margin; DFS: disease-free survival; IHC: immunohistochemical; NGS: next generation sequencing; OS: overall survival).

From a total cohort of 283 patients who received surgery for hPDAC, 55 patients met our predefined inclusion criteria for NGS. Figure 1 represents the flow chart of the study. Tumor areas with ≥80% tumor cellularity were dissected for DNA extraction, thus minimizing stromal contamination. In 16 patients, no adequate DNA sequencing was possible because of low quantities or insufficient quality of DNA. The analysis was performed in the remaining 39 patients (Table 1 and Figure 1). 

The median age of all 39 patients at the time of surgery was 69 years (range: 47–90). Our cohort consisted of 24 male (61.5%) and 15 female (38.5%) patients. All patients received a partial pancreaticoduodenectomy. Thirty-three patients (84.62%) received gemcitabine monotherapy, while six patients (15.38%) received a combination therapy with paclitaxel. FOLFIRINOX as a standardized adjuvant treatment regime was not applied to our study cohort based on the guidelines at the time the study was performed. Follow-up data were available for all patients. A total of 25 patients (64.1%) died during the follow-up period. The median OS of all 39 patients was 18.8 months (range: 2.16 months–154.4 months). The median DFS was 7.92 months (range: 1.2 month–154.4 months). Of the 39 patients, 26 patients were diagnosed with metachronous relapse during follow-up (66.7%). In 11 patients, metachronous hepatic metastases were evident, whereas in 12 and 3 patients, pulmonary relapse and peritoneal carcinomatosis were diagnosed, respectively. The median RSS for the 26 patients was 7.03 months (range: 0–36.2 months).

Cases were reclassified into histomorphological subtypes according to current criteria. In thirty-one (79.5%) patients, the tumor was classified as classical PDAC, in four patients as anaplastic, and in a further four patients each as papillary, medullary, complex and large-duct type PDAC, respectively. Additional immunohistochemical staining of tumor slides was successfully performed for p53 in 35 patients, for p16 in 36 patients, and for Smad4 in 37 patients, respectively. Aberrant immunohistochemical expression of p53 was found in 16 patients (20/35, 57.1%). Loss of immunohistochemical expression of p16 and Smad4 was found in 26 (26/36, 72.2% for p16 and 26/37, 70.3% for Smad4). A correlation analysis was performed between histomorphological subgroups (classical PDAC vs. rest), immunohistochemical staining results (p53, p16 and Smad4) and clinicopathological variables (Supplemental Tables S2 and S3). When patients were stratified according to histomorphology (classical PDAC vs. rest), all incorporated clinicopathological variables were homogenously distributed (Supplemental Table S2). A distribution analysis was also performed regarding immunohistochemical results (aberrant expression of p53, p16 and Smad4) (Supplemental Table S3). Only patients with an aberrant expression of p16 showed a higher T category when compared to patients without aberrant p16 expression (*p* = 0.011).

### 3.2. Panel Next Generation Sequencing

**Figure 2:**
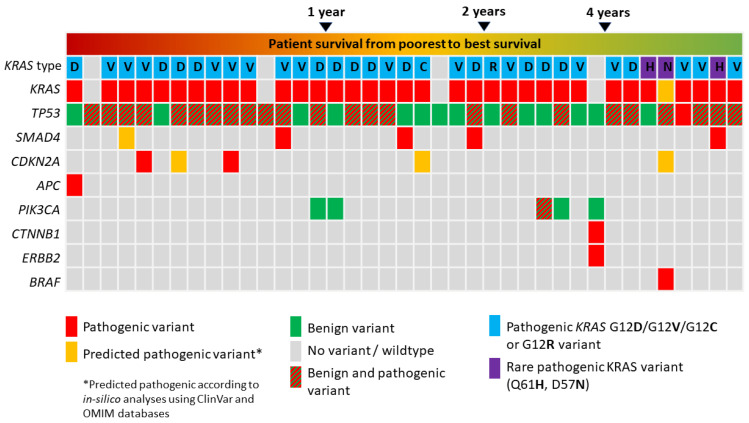
Oncoprint with an overview of all cases in this study whose next generation panel sequencing (NGS) was successfully performed. Rare pathogenic mutations of *KRAS* were only noted in long-term survivors.

Results of mutation analysis are summarized in Figure 2 and Supplemental Table S4. From the total study collective, pathogenic *KRAS* mutations were found in 35 patients (89.74%). In 16 patients (41.02%), *KRAS* missense mutations resulted in the amino acid change G12V. Fourteen patients (35.90%) harbored *KRAS* missense mutations with resulting the amino acid change G12D, one *KRAS* mutation resulted in the amino acid change G12C and one further patient harbored a *KRAS* missense mutation with the resulting amino acid change G12R (each 2.8%). In three patients (10.26%), rare *KRAS* missense mutations were evident (two patients with Q61H, one patient with D57N alteration). Twenty-five (64.10%) patients harbored pathogenic *TP53* mutations. Missense mutations were evident in 20 patients (51.28%), whereas nonsense and frameshift/splice-site mutations were evident in 5 patients (12.8%) (Supplemental Table S4).

An additional third driver mutation was present in 13 patients (33.33%) (Supplemental Table S4). Of these 13 patients, five (38.46%) harbored *SMAD4* mutations, followed by five patients (38.46%) with *CKDN2A*, (one patient with an additional *BRAF* mutation and one patient with an additional *ATM* mutation), and three patients with an *APC*, *CTNNB1/ERBB2* or *PIK3CA* mutation, respectively (each 7.69%). In 13 (92.86%) and 9 (64.29%) patients, an additional *KRAS* and *TP53* mutation was found, respectively. In the correlation analysis, patients with or without *KRAS* and *TP53* mutations showed similar tendencies for a further driver mutation (Supplemental Tables S3 and S4). A distribution analysis was performed between *KRAS*, *TP53* and the third driver mutational status and the following variables: clinicopathological variables, histomorphology and immunohistochemical expression status (Table 2). All incorporated variables were homogenously distributed in patients, independently from the *KRAS* and *TP53* mutational status as well as in patients with or without further driver mutations (Table 2).

### 3.3. Allele Frequency Rates of Pathogenic KRAS and TP53 Variants

The allele frequency rates were analyzed in the 35 patients with pathogenic *KRAS* mutations and in the 25 patients with pathogenic *TP53* mutations. The median allele frequency rate of pathogenic *KRAS* mutations was 12% (range: 2–32%), while the median allele frequency rate of pathogenic *TP53* mutations was 14% (range: 2–66%).

The median allele frequency rate of *KRAS* variants in patients with *KRAS* G12D mutation was higher when compared to patients with other *KRAS* mutations, but the difference did not reach statistical significance (*p* = 0.854). In patients with nonsense and frameshift/splice-site mutations of *TP53* and in patients with missense mutations of *TP53*, the median allele frequency rate of pathogenic *TP53* mutations was 14% and without statistical significance (*p* = 0.705). Patients with poorly differentiated (G3) PDACs had higher median allele frequency rate of pathogenic *TP53* mutations when compared to patients with G1/G2 PDACs (*p* = 0.029). 

All other analyzed subgroups (e.g., depending on histomorphology, immunohistochemical profile or stage) did not show significant differences in the allele frequency of *KRAS* and *TP53* mutations (Supplemental Table S5).

### 3.4. Survival Analysis

**Figure 3:**
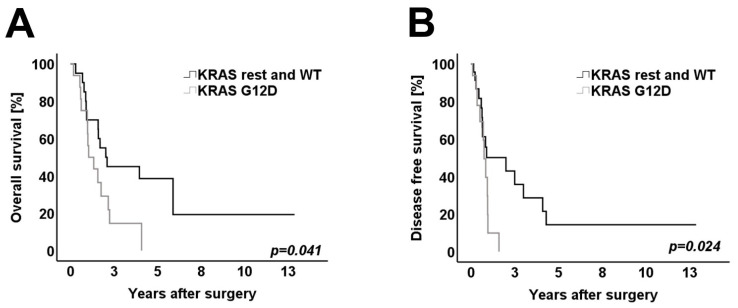
Kaplan–Meier survival curves for: (**A**) Overall survival of patients with a *KRAS* G12D mutation and patients with other *KRAS* mutations and WT (*n* = 39). (**B**) Disease-free survival of patients with *KRAS* G12D mutation and patients with other KRAS mutation and WT (*n* = 39). Log-rank test was used to test for significance. *p*-value < 0.05 indicates significance. Patients with *KRAS* G12D mutations showed a significantly worse OS and DFS when compared to patients with all other *KRAS* mutations or WT.

Overall survival, disease-free survival and relapse-specific survival were analyzed for all 39 patients. First, a univariate survival analysis was performed considering all studied clinicopathological variables. None of the studied variables correlated significantly with worse overall survival. For the survival analysis of *KRAS* mutational status, a subgroup analysis was performed. Patients with a *KRAS* G12D mutation (*n* = 14) were compared to patients with *KRAS* wild-type or other pathogenic *KRAS* mutations (*n* = 25). Clinicopathological variables and histomorphology were distributed homogenously between both *KRAS* subgroups. In the univariate analysis, patients with a *KRAS* G12D mutation showed significantly worse OS and DFS when compared to patients with *KRAS* wild-type and patients with different pathogenic *KRAS* mutations (*p* = 0.041 for OS and *p* = 0.024 for DFS) (Figure 3A,B). 

**Figure 4:**
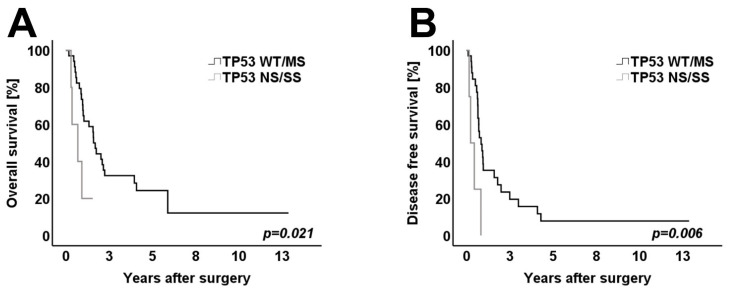
Kaplan–Meier survival curves for (**A**) overall survival of patients and (**B**) disease-free survival of patients with NS/FS/SS *TP53* mutations and patients with MS mutations of *TP53* or WT (*n* = 39). Log-rank test was used to test for significance. *p*-value < 0.05 indicates significance. Patients with *TP53* nonsense and splice-site mutations showed a significantly worse OS and DFS survival when compared to patients with TP miss-sense mutations or WT.

**Figure 5:**
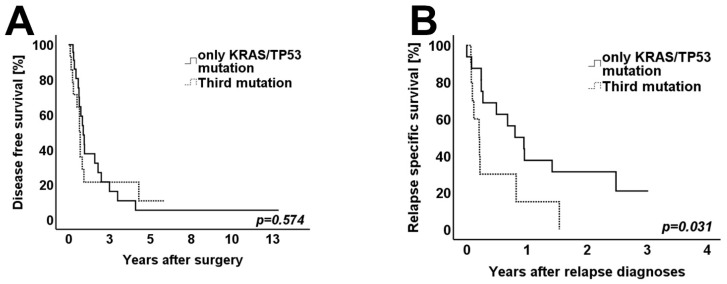
Kaplan–Meier survival curves for: (**A**) Disease-free survival of patients with any third mutation and patients harboring *KRAS* mutation and/or *TP53* mutations (*n* = 39). (**B**) Relapse-specific survival of patients with any third mutation and patients harboring *KRAS* mutation and/or *TP53* mutations (*n* = 39). Log-rank test was used to test for significance. *p*-value < 0.05 indicates significance.

The *TP53* mutational status was subgrouped into *TP53* nonsense and frameshift/splice-site mutations (*n* = 5) vs. *TP53* wild-type or *TP53* missense mutations (*n* = 34). Again, clinicopathological variables and histomorphology were distributed homogenously between both groups. In the univariate analysis, patients with nonsense, frameshift/splice-site *TP53* mutations showed a significantly worse OS and DFS when compared to patients with *TP53* wild-type or *TP53* missense mutations (*p* = 0.021 for OS and *p* = 0.006 for DFS) (Figure 4A,B). Patients with a third driver mutation other than *KRAS* and *TP53* showed a similar DFS when compared to patients harboring only either *KRAS* and/or *TP53* mutations (*p* = 0.574) (Figure 5A). However, when considering the 26 patients with metachronous relapse, patients with a third driver mutation showed a significantly worse RSS when compared to patients in which only *KRAS* and *TP53* mutations were detected (*p* = 0.031) (Figure 5B). 

Subgroup analysis was performed between short-term and long-term overall survivors and short-term and long-term disease-free survivors (Table 3). Again, clinicopathological and histomorphological variables were distributed homogenously between groups. *KRAS* G12D mutation, nonsense and frameshift/splice-site mutations of *TP53*, evident third or *SMAD4* mutations were solely or mostly found in short-term overall or disease-free survivors (<4 years), whereas rare the *KRAS* mutations Q61H and D57N were only found in long-term overall or disease-free survivors (*p* = 0.015 and *p* = 0.029) (Table 3). 

**Figure 6:**
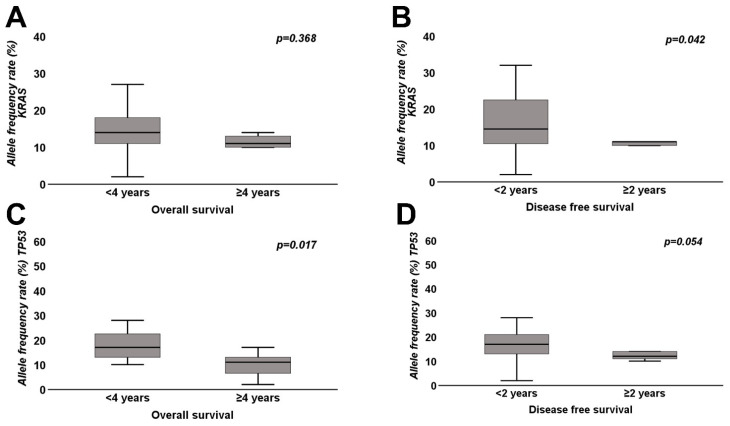
Box plots of (**A**) *KRAS* allele frequency rate and overall survival (*n* = 39). (**B**) *KRAS* allele frequency rate and disease-free survival (*n* = 39). (**C**) *TP53* allele frequency rate and overall survival (*n* = 39). (**D**) *TP53* allele frequency rate and disease-free survival (*n* = 39). Mann–Whitney U tests were used to test for significance (*p*-value < 0.05 indicates significance).

A correlation analysis of median allele frequency rates of pathogenic *KRAS* and *TP53* mutations in overall and disease-free survival subgroups was performed (Table 4, Figure 6A–D)**.** The median allele frequency rates of pathogenic *KRAS* and *TP53* was higher in poor overall and disease-free survivors. The higher median allele frequency rate of *KRAS* reached statistical significance only in poor disease-free survivors, whereas the higher median allele frequency rate of *TP53* reached statistical significance in poor overall survivors (Table 4, Figure 6A–D). 

## 4. Discussion

PDAC is an extremely aggressive malignancy with a five-year survival rate that has been stagnant around 10% for decades despite substantial research efforts regarding early diagnosis, adequate patient stratification and improvement of therapy. The concept of PDAC as a uniform disease has already been abandoned, as not only distinct histomorphological subtypes, but also molecular subtypes of PDAC have emerged in studies using a high-throughput molecular analysis of PDAC [28,29,30,31]. The proposed molecular and histomorphological subtypes seem to be linked to patient survival and, in part, to therapy response [28,29,30,31]. However, molecular subtyping of PDAC is still in need of validation and further optimization. For example, the molecular subtypes proposed by different authors show certain similarities, but do not overlap perfectly. In addition, the molecular and histomorphological subtypes also only correspond partially [17].

In this study, we aimed for a thorough histomorphological, molecular and survival analysis of PDAC in a clinically and pathologically well-characterized cohort of patients after a multimodal therapy for hPDAC, with focus on short- vs. long-term survival of pancreatic cancer. 

Pathological staging variables as well as histomorphological subgroups were evenly distributed between short- and long-term survivors in this study. Interestingly, the G12D *KRAS* mutation, nonsense/frameshift/splice-site *TP53* mutation, *SMAD4* mutation or any other third mutations were solely present in short-term survivors. On the other hand, two rare *KRAS* mutations, Q61H and D57N, were found only in long-term survivors. As the tumor cellularity was carefully assessed before DNA isolation, we were also able to compare the allele frequency rates of pathogenic *KRAS* and *TP53* mutations. *KRAS* mutations showed a significantly higher median allele frequency rate in patients with shorter DFS when compared to patients with a longer DFS, and *TP53* mutations showed a significantly higher median allele frequency rate in patients with a shorter OS when compared to patients with a longer OS. 

Genome-based sequencing of resected PDACs has already been performed extensively in the past and revealed undisputedly that *KRAS*, *TP53* and *SMAD4* are the most commonly mutated genes in PDAC [32,33,34,35]. However, data regarding the question whether the mutational profile of short-term and long-term survivors of pancreatic cancers is significantly different, especially regarding the aforementioned mutations, are inconclusive. While Masetti et al. proposed that long-term survivors of PDAC generally show fewer mutations of *KRAS*, *TP53* and *SMAD4*, Dal Molin and colleagues suggested that the mutational status of long-term survivors’ PDACs does not significantly differ from PDACs unselected for survival [16,32]. These differences in results may be due to methodological limitations of the studies. While in the study by Masetti et al. clinicopathological variables were heterogeneously distributed among patients, Dal Molin et al. did not include a group of PDAC short-term survivors in their analysis. In a more recent study, Yokose et al. suggested that a combination of *KRAS* and *SMAD4* mutations is an independent prognostic factor indicating short-term-survival of pancreatic cancer. However, in this study, adjuvant therapy was only administered in 84% of the patients, only 56% of the patients had PDAC of the pancreatic head, and the R status was not evaluated according to otherwise well-established standardized protocols, resulting in limited comparability of the data [36,37,38]. 

*KRAS* mutations in PDAC are known to act as the driver for tumor formation. Studies have shown that the presence of specific *KRAS* mutations is influenced by the mutagenic events taking place in the tissue. For example, in PDAC, the most frequently observed pathogenic variant, the G12D *KRAS* mutation (the replacement of nucleotide sequence GGT with GAT in codon 12 of exon 2 of the *KRAS* gene), is linked to a clock-like mutational event (intrinsic, related to the patient’s age), while Q61H *KRAS* mutations are linked to oxidative stress [39]. In turn, different pathogenic *KRAS* mutations result in alternating activation cascades in the *KRAS* pathway [39]. It has been shown that the prognostic significance of a *KRAS* mutation is dependent on which specific *KRAS* mutation is present and thus, which activation cascade is activated [40]. A G12D *KRAS* mutation is generally associated with worse survival, e.g., when compared to *KRAS* mutations in codon 61, which, in turn, indicate a favorable prognosis [40,41]. Our study showed similar findings. Mutations resulting in subtype G12D were also the most frequent in our collective and were associated with worse OS and DFS. On the other hand, a Q61H mutation of *KRAS* was found only in two cases in our cohort, and these were cases of long-term PDAC survivors.

In addition to which specific pathogenic *KRAS* mutation was present, patient survival seemed to depend on the allele frequency of pathogenic *KRAS* mutations in our cohort: patients with higher allele frequency rates of pathogenic *KRAS* mutations showed significantly poorer DFS than patients with lower allele frequency rates of pathogenic *KRAS* mutations. The allele frequency in somatic tumor samples is dependent on several factors, most prominently on the presence of somatic vs. germline mutation and the proportion of tumor cells within the sample (tumor cellularity) [42]. As PDAC is driven by somatic rather than germline *KRAS* mutations and the tumor cellularity was ensured to be sufficient and comparable in all samples used in this study, we may hypothesize that the allele frequency may have at least some predictive value regarding the proportion of tumor cells carrying a mutation of the respective gene (i.e., percentage of tumor cell subclones with a pathogenic *KRAS* mutation) in this cohort. These different median allele frequency rates of pathogenic *KRAS* mutations between short- and long-term PDAC survivors might be explained by the role of *KRAS* mutations in inducing tumor progression, with tumors showing higher allele frequency rates for pathogenic *KRAS* mutations, i.e., tumors harboring a higher proportion of *KRAS*-mutated subclones, showing a faster tumor progression, hence its link to early disease recurrence in our study. The impact of the allele frequencies of pathogenic mutations on patient outcome has already been evaluated in other solid malignancies, e.g., in malignant melanoma [43].

*TP53* prevents cancer formation and thus functions as a tumor suppressor gene and represents one of the most common genetic lesions in cancer [44,45]. We noticed that in 64% of cases in our study cohort, pathogenic *TP53* mutations were evident. More importantly, nonsense and frameshift/splice-site mutations were solely evident in patients with a short OS and DFS, corroborating previously published results linking pathogenic mutations of *TP53* to a shortened disease-free survival of pancreatic cancer [44]. The phenotypical effects of mutant *TP53* in pancreatic cancer were demonstrated by Weissmueller et al. in an in vitro analysis, which showed that the sustained expression of the mutant *TP53* allele is necessary to maintain the invasive phenotype of PDAC [46]. Although *TP53* mutations have been suggested to play a role in a worse survival of PDAC patients, pathogenic alterations of *TP53* may also be associated with gemcitabine sensitivity [44].

A loss of function of *SMAD4* is known to correlate with a significantly greater chance of metastatic relapse and with overall worse prognosis [47,48]. Our data revealed similar findings since *SMAD4* mutations were almost exclusively found in short-term survivors of PDAC. In addition, patients with three different driver mutations of the primary tumor, including a loss of function of *SMAD4,* showed a significantly worse relapse-specific survival, confirming the results of previous studies [17].

This study has several limitations. NGS analysis was only performed in a small fraction of cases. Thus, only a univariate survival analysis could be performed. Nevertheless, all survival-relevant clinicopathological variables such as T category, N category, and tumor grading, were evenly distributed across *KRAS*, *TP53* and third driver mutation subgroups. Furthermore, DNA sequencing was not feasible in many paraffin-embedded tumor tissues stored for a long time (>2 years). Thus, a lower number of long-term survivors were evaluated compared to patients who succumbed earlier to disease progression. Hence, the future focus should be placed on prospective multicenter studies to identify long-term survivors of pancreatic cancer in order to understand mutational differences that clearly separate short-term vs. long-term survivors. 

## 5. Conclusions

In summary, our results suggest that somatic variants in genes commonly mutated in PDAC are presumably primary determinants for superior DFS and OS in PDAC patients. Because long-term survival of surgically resected PDAC is rare, results from a single institution analysis should be validated. Multi-institutional studies are clearly warranted in order to understand mutational differences, and, if available in the future, to apply targeted therapies. 

## Figures and Tables

**Table 1:** Demographic data of the patient cohort from 2004–2016; *n* = 39. Most patients were diagnosed with an advanced stage of disease.

Age in Years	Patients (Total)	Patients (Percentage)
**Median (Range)**	66 (47–88)	
	***n***	**%**
**Gender**		
Male	24	61.5
Female	15	38.5
**T stage**		
T1	3	7.7
T2	18	46.2
T3	16	41.0
T4	2	5.1
**N stage**		
N0	7	17.9
N1	23	59.0
N2	9	23.1
**Grading**		
G2	27	69.2
G3	12	30.8
**Pn**		
Pn0	10	25.6
Pn1	29	74.4
**L**		
L0	16	41.0
L1	23	59.0
**V**		
V0	27	69.2
V1	12	30.8
**R status**		
R1	16	41.0
R0CRM+	6	15.4
R0CRM−	17	43.6

CRM: circumferential resection margin; Pn: perineural invasion; L: lymphatic invasion; V: venous invasion.

**Table 2:** Correlation analysis between clinicopathological variables and mutations. Chi-squared test (*p*-value < 0.05 indicates significance). There was a homogenous distribution of clinicopathological, histomorphological and immunohistochemical variables across mutation subgroups.

	NGS KRAS G12D vs. Rest and WT	NGS TP53 NS/FS/SS vs. Rest and WT	3rd Mutation vs. Only KRAS/TP53 Mutation
	*n* = 39	*n* = 39	*n* = 39
Chi-squared test	*p*-value	*p*-value	*p*-value
**Median Age**	0.471	0.823	1.000
**Gender**	0.944	0.401	0.740
**T stage**	0.921	0.887	0.337
**N stage**	0.921	0.669	0.864
**G**	0.288	0.239	0.824
**Pn**	0.326	0.066	0.052
**L**	0.492	0.801	0.510
**V**	0.609	0.535	0.728
**Resection status**	0.275	0.951	0.855
**Relapse location**	0.114	0.184	0.733
**Histomorphology**	0.673	0.533	0.643
**p53 IHC**	0.561	0.369	0.489
**p16 IHC**	0.924	0.625	0.716
**Smad4 IHC**	0.161	0.714	0.143

IHC: immunohistochemistry; L: lymphatic invasion; NGS: next generation sequencing; NS/FS/SS: nonsense/frameshift/splice-site; Pn: perineural invasion V: venous invasion, WT: wild-type.

**Table 3:** Correlation analysis between survivor subgroups and oncogenic mutation; *n* = 39. Fisher’s exact test was used to test for significance (*p*-value < 0.05 indicates significance).

Mutational Status	OS	OS		DFS	DFS	
	<4 Years	≥4 Years	*p*-Value	<2 Years	≥2 Years	*p*-Value
	*n* = 31	*n* = 8		*n* = 32	*n* = 7	
**KRAS NGS**						
G12D	13	1	**0.015**	14	0	**0.005**
G12V/R/C and WT	18	4		18	4	
Q61H/D57N	0	3		0	3	
**TP53 NGS**						
NS + SS	5	0	0.563	5	0	0.563
WT + MS	26	8		27	7	
**SMAD4 NGS**						
Yes	4	1	1.000	5	0	0.898
No	27	7		-	-	
**Other 3rd driver mutation**						
Yes	11	2	0.751	11	2	0.884
No	20	6		21	5	
**p53 IHC**						
Aberrant	15	5	1.000	16	4	1.000
Normal	12	3		12	3	
**p16 IHC**						
Normal	8	2	1.000	8	2	1.000
Loss	20	6		21	5	
**Smad4 IHC**						
Normal	8	3	0.672	7	4	0.163
Loss	21	5		23	3	

DFS: disease-free survival; IHC: immunohistochemistry; NGS: next generation sequencing; NS: nonsense; MS: missense; OS: overall survival; SS: splice site; WT: wild type.

**Table 4:** Correlation analysis between survivor subgroups and median allele frequency rates of pathogenic *KRAS* and *TP53* mutations. A Mann–Whitney U test was used to test for significance (*p*-value < 0.05 indicates significance).

	OS	OS		DFS	DFS	
	<4 Years	≥4 Years	*p*-Value	<2 Years	≥2 Years	*p*-Value
	*n* = 31	*n* = 8		*n* = 32	*n* = 7	
**Median (range)** **allele** **frequency rate** ***KRAS***	14% (2–32%)	11% (4–30%)	0.368	14.5% (2–32%)	11.0% (4–13%)	**0.042**
**Median (range)** **allele** **frequency rate** ***TP53***	17% (10–54%)	11% (2–17%)	**0.017**	17% (2–54%)	12% (10–14%)	0.054

DFS: disease-free survival; OS: overall survival.

## Data Availability

The datasets used and/or analyzed during the current study are available from the corresponding author on reasonable request.

## Supplementary Materials

The following are available online at https://www.mdpi.com/article/10.3390/cancers14030850/s1, Table S1: Primary antibodies used for immunohistochemistry. Table S2: Correlation analysis between immunohistochemical expression status of p53, p16 and Smad4 and clinicopathological variables. A Mann–Whitney U test was used to test for significance (*p*-value < 0.05 indicates significance); Table S3: Correlation analysis between clinicopathological variables, immunohistochemical expression status of p53, p16 and Smad4 and histomorphology (classical vs. rest). A Mann–Whitney U test was used to test for significance (*p*-value < 0.05 indicates significance); Table S4: Correlation analysis between *KRAS* and *TP53* mutations; n = 39. Statistical significance was calculated by a Mann–Whitney U test and Fisher’s exact test (*p*-value < 0.05 indicates significance). The distribution of different mutations across *KRAS* and *TP53* was similar. Correlation analysis between third mutations, *KRAS* and *TP53* mutations; n = 39. There was a homogenous distribution between *KRAS* and *TP53* mutations and third evident mutation. The statistical significance was calculated by a Mann–Whitney U test and Fisher’s exact test (*p*-value < 0.05 indicates significance); Table S5: Correlation analysis between *KRAS*/*TP53* allele frequency rate and histomorphological as well as clinicopathological subgroups. A Mann–Whitney U test was used to test for significance (*p*-value < 0.05 indicates significance). Supplemental Figure S1. Histomorphology of an exemplary PDAC case from the present patient cohort. An area with adequate tumor cellularity (≥80%) was marked for manual macrodissection (yellow dotted line) (H&E, 30×); Supplemental Figure S2. Immunohistochemical (IHC) analyses. (A–C): IHC of p53. Wild-type expression pattern with staining of weak intensity in some of the tumor cell nuclei (A), aberrant expression in the form of overexpression (B) and aberrant expression in the form of complete loss of expression are shown (100×, respectively). (D,E) IHC of Smad4. Wild-type expression pattern with nuclear positivity of Smad4 in PDAC cells (D) and aberrant expression in the form of complete loss of Smad4 expression in PDAC cell nuclei (E) are shown (100×, respectively). (F,G) IHC of p16. Wild-type expression pattern with cytoplasmic and nuclear p16 positivity of PDAC cells (F, 100×) and aberrant expression in the form of complete loss of p16 expression in PDAC cells (G, 200×) are shown. In (E) and (G), Smad4- and p16-positive stromal cells serve as internal positive controls, respectively.
