# Supplementary material for: Genetic Alterations Predict Long-Term Survival in Ductal Adenocarcinoma of the Pancreatic Head
Source: Cancers (Basel). 2022 Feb 8;14(3):850. doi: 10.3390/cancers14030850 (PMC8833892; doi:10.3390/cancers14030850)
Supplement: Supplementary file 1 [file cancers-14-00850-s001.zip › Supplementals final_NEW/Supplementals final_NEW.pdf]

Table S1: Primary antibodies used for immunohistochemistry.

Antibody	Manufacturer	Clone (animal)	Dilution
Anti-$p16^{INK4A}$	Diagnostic BioSystems (Pleasanton, CA, USA)	Clone JC2 (mouse)	1:200
Anti-p53	Calbiochem (San Diego, CA, USA)	Clone DO-1 (mouse)	1:200
Anti-Smad4	Zeta Corporation (Arcadia, CA, USA)	Clone B-8 (mouse)	1:50

Table S2: Correlation analysis between immunohistochemical expression status of p53, p16 and Smad4 and clinicopathological variables. Mann-Whitney U test was used to test for significance (*p*-value < 0.05 indicates significance).

	Histomorphology<br>Classical vs. rest<br>Mann-Whitney-U test<br>***p*-value**
**Gender** (female vs. male)	*0.157*
**T stage** (T1/T2 vs. T3/T4)	*0.207*
**N stage** (N0 vs. N1/N2)	*0.928*
**Grading** (G2 vs. G3)	*0.099*
**Pn** (Pn0 vs. Pn1)	*0.849*
**L** (L0 vs. L1)	*0.354*
**V** (V0 vs. V1)	*0.293*
**p53** (aberrant vs. normal)	*0.180*
**p16** (normal vs. negative)	*0.290*
**Smad4** (normal vs. negative)	*0.308*

Pn: perineural invasion; L: lymphatic invasion; V: venous invasion

Table S3: Correlation analysis between clinicopathological variables, immunohistochemical expression status of p53, p16 and Smad4 and histomorphology (classical vs. rest). Mann-Whitney U test was used to test for significance (p-value < 0.05 indicates significance).

	p53	p16	Smad4
		Mann-Whitney-U test	
	***p*-value**	***p*-value**	***p*-value**
**Histomorphology** (classical vs. rest)	*0.483*	*0.539*	*0.986*
**Gender** (female vs. male)	*0.564*	*0.931*	*0.781*
**T stage** (T1/T2 vs. T3/T4)	*0.633*	***0.011***	*0.883*
**N stage** (N0 vs. N1/N2)	*0.633*	*0.903*	*0.806*
**Grading** (G2 vs. G3)	*0.882*	*0.227*	*0.731*
**Pn** (Pn0 vs. Pn1)	*0.560*	*0.733*	*0.892*
**L** (L0 vs. L1)	*0.202*	*0.872*	*0.813*
**V** (V0 vs. V1)	*0.681*	*0.733*	*0.685*

Pn: perineural invasion; L: lymphatic invasion; V: venous invasion

Table S4: Correlation analysis between *KRAS* and *TP53* mutations; $n = 39$. Statistical significance was calculated by Mann-Whitney U test and Fisher's exact test (p-value < 0.05 indicates significance). Distribution of different mutations across *KRAS* and *TP53* was similar. Correlation analysis between 3rd mutations, *KRAS* and *TP53* mutations; $n = 39$. There was a homogenous distribution between *KRAS* and *TP53* mutations and 3rd evident mutation. Statistical significance was calculated by Mann-Whitney-U test and Fisher's exact test (p-value < 0.05 indicates significance).

	**KRAS mutation NGS**							***p*-value**
	**G12D**	**G12V**	**G12R**	**G12C**	**Q61H**	**D57N**	**WT**	
**TP53 mutation**								*0.740*
**WT**	6	3	1	1	1	0	2	
**NS**	1	1	0	0	0	0	0	
**FS/SS**	0	3	0	0	0	0	0	
**MS**	7	9	0	0	1	1	2	

	**3rd driver mutations**								***p*-value**
	**No**	**APC**	**ATM**	**BRAF**	**CDKN2A**	**CTNNB1/ERBB2**	**PIK3CA**	**SMAD4**	
**TP53 mutation**									*0.580*
**WT**	9	1	1*	0	1*	1	1	1	
**NS**	1	0	0	0	0	0	0	1	
**FS/SS**	2	0	0	0	0	0	0	1	
**MS**	14	0	0	1*	4*	0	0	2	
**KRAS mutation**									*0.915*
G12D	9	1	0	0	1	0	1	2	
G12V	12	0	0	0	2	0	0	2	
G12R	1	0	0	0	0	0	0	0	
G12C	0	0	1*	0	1*	0	0	0	
Q61H	1	0	0	0	0	0	0	1	
D57N	0	0	0	1*	1*	0	0	0	
WT	3	0	0	0	0	1	0	0	

*FS/SS: frameshift/splice-site; NGS: next generation sequencing; NS: non-sense; MS: miss-sense; WT: wild-type; *: one patient*

Table S5: Correlation analysis between *KRAS*/*TP53* allele frequency rate and histomorphological as well as clinicopathological subgroups. Mann-Whitney-U test was used to test for significance (*p*-value < 0.05 indicates significance).

	Mutational frequency rate	
	KRAS	TP53
	Mann-Whitney-U test	
	***p*-value**	***p*-value**
**Histomorphology (**classical vs. rest)	*0.254*	*0.574*
**Gender** (female vs. male)	***0.011***	*0.434*
**T stage** (T1/T2 vs. T3/T4)	*0.258*	*0.211*
**N stage** (N0 vs. N1/N2)	*0.398*	*0.152*
**Grading** (G2 vs. G3)	*0.630*	***0.029***
**Pn** (Pn0 vs. Pn1)	*0.500*	*0.061*
**L** (L0 vs. L1)	*0.504*	*0.697*
**V** (V0 vs. V1)	*0.619*	*0.082*
**p53** (Aberrant vs. Normal)	*0.668*	*0.560*
**p16** (Normal vs. Loss)	*0.499*	*1.000*
**Smad4** (Normal vs. Loss)	*0.707*	*0.640*

Pn: perineural invasion; L: lymphatic invasion; V: venous invasion

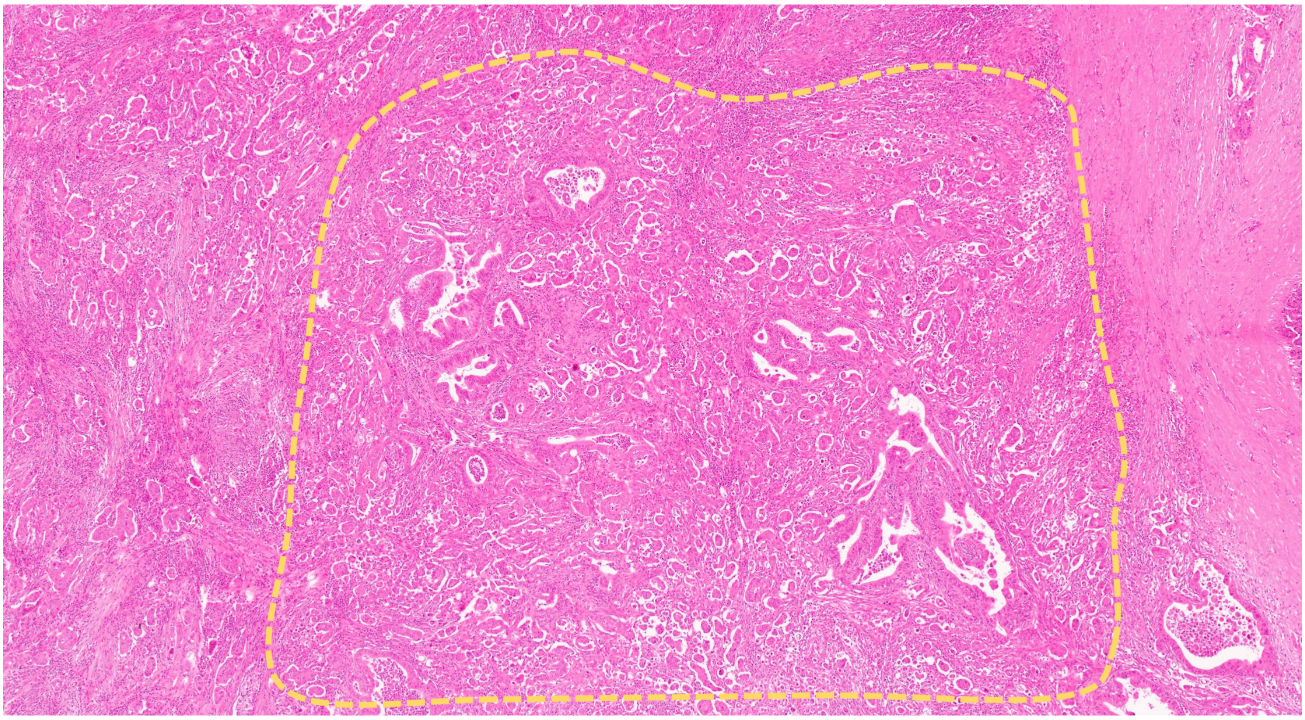

Figure S1. Histomorphology of an exemplary PDAC case from our cohort. An area with adequate tumor cellularity (≥ 80%) was marked for manual macrodissection (yellow dotted line) (H&E, 30x).

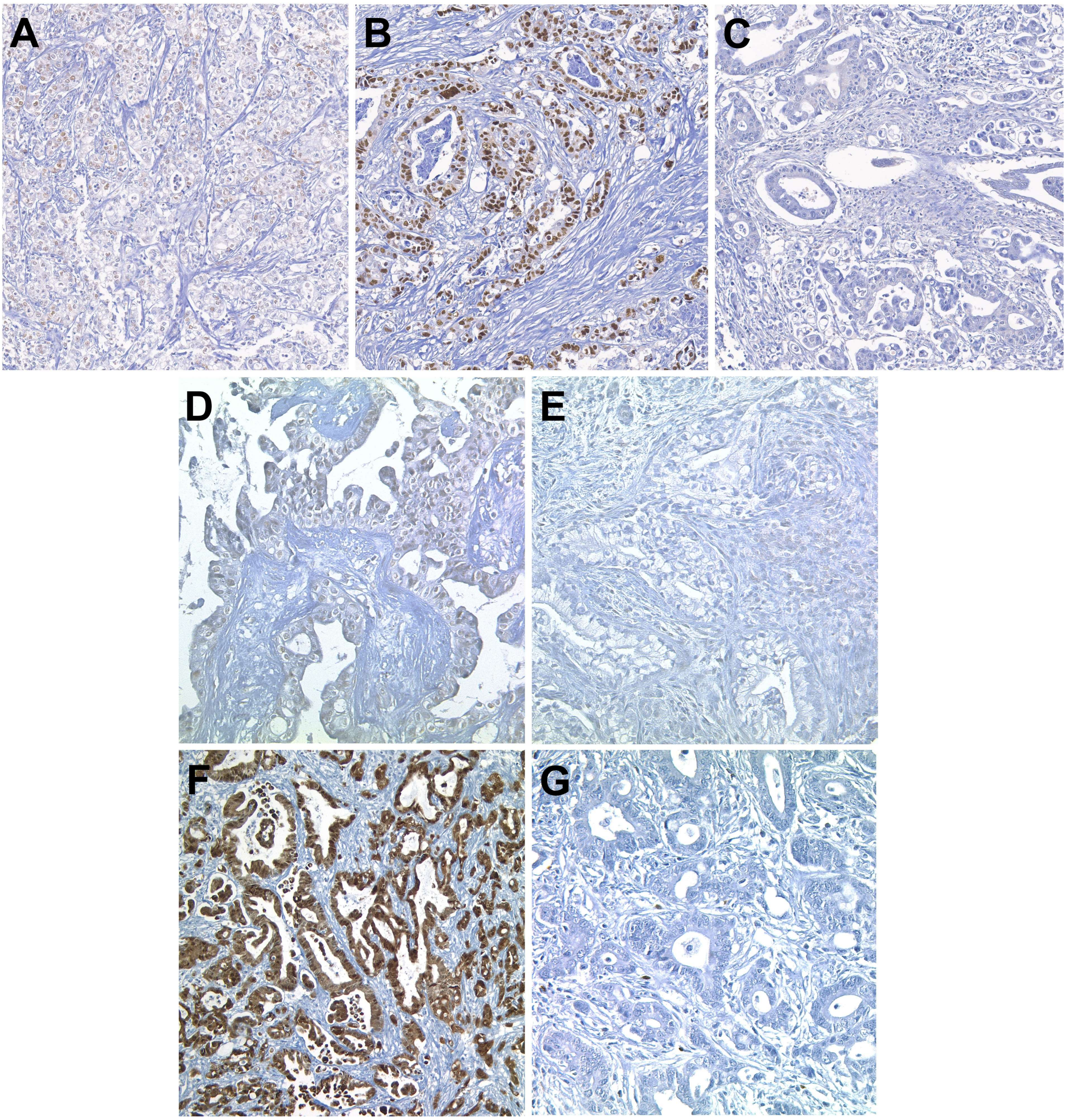

S2. Immunohistochemical (IHC) analyses. A-C: IHC of p53. Wild-type expression pattern with staining of weak intensity in some of the tumor cell nuclei (A), aberrant expression in the form of overexpression (B) and aberrant expression in the form of complete loss of expression are shown (100x, respectively). **D & E.** IHC of Smad4. Wild-type expression pattern with nuclear positivity of Smad4 in PDAC cells (D) and aberrant expression in the form of complete loss of Smad4 expression in PDAC cell nuclei (E) are shown (100x, respectively). **F & G.** IHC of p16. Wild-type expression pattern with cytoplasmic and nuclear p16 positivity of PDAC cells (F, 100x) and aberrant expression in the form of complete loss of p16 expression in PDAC cells (G, 200x) are shown. In E. and G., Smad4- and p16-positive stromal cells serve as internal positive controls, respectively.
